# Neonatal sepsis and its association with birth weight and gestational age among admitted neonates in Ethiopia: systematic review and meta-analysis

**DOI:** 10.1186/s12887-020-1949-x

**Published:** 2020-02-05

**Authors:** Amare Belachew, Tilahun Tewabe

**Affiliations:** College of Medicine and Health Science, Bahir Dar University, Bahir Dar, Ethiopia

**Keywords:** Neonatal sepsis, Prevalence, Determinants, Newborn, Ethiopia

## Abstract

**Background:**

Neonatal sepsis is an invasive infection, usually bacterial, and often occurring during the neonatal period (0–28 days). Neonatal sepsis causes a high burden of morbidity and mortality in developing countries like Ethiopia. There are fragmented, inconsistency, and no review has been conducted to report the magnitude and associated factors of neonatal sepsis in Ethiopia. Thus, this study aimed to assess the pooled prevalence of neonatal sepsis and its association with birth weight and gestational age among admitted neonates in Ethiopia.

**Methods:**

Electronic media searches like PubMed, CINHAL, EMBASE, Google Scholar, Web of Science, Cochrane library databases and African health science library were used. All original peer-reviewed papers which reported the prevalence of neonatal sepsis in Ethiopia were included in this study. Two reviewers independently extracted the data using a standardized data extraction format for eligibility and appraised their quality. Data were analyzed using Stata version 14 software. The pooled prevalence of neonatal sepsis was estimated with the random-effect model. Heterogeneity between studies was assessed by *I* ^*2*^ statistics test. Subgroup and meta-regression analyses were done to assess the source of variation between the studies. Egger’s test followed by trim and fill analysis were used to determine publication bias. A sensitivity analysis was carried out.

**Result:**

A total of 952 research papers reviewed, of which, eight studies were finally included in this systematic review and meta-analysis. The random effect pooled prevalence of neonatal sepsis in Ethiopia was 49.98% (CI: 36.06, 63.90). In subgroup analysis, the pooled estimated neonatal sepsis among cross-sectional studies was 53.15% while the cohort was 40.56%. Newborns with a birth weight of less than 2.5 kg were 1.42 times more likely to develop neonatal sepsis infection compared to normal babies. The odds ratios of preterm babies were 3.36 to develop neonatal sepsis compared to term infants.

**Conclusion:**

The pooled prevalence of neonatal sepsis in Ethiopia was high. Thus, health care providers should adhere to aseptic precautions while performing procedures, especially in preterm and low birth weight infants were recommended.

## Background

Neonatal sepsis is a bloodstream infection. It is a major cause of morbidity and mortality for newborns. It can be categorized as early onset or late onset neonatal sepsis. Of newborns with early onset sepsis, 85% present within 24 h, 5% present at 24–48 h, and a smaller percentage present within 48–72 h [1]. Early onset neonatal sepsis is associated with the acquisition of microorganisms from the mother, birth canal during delivery [1].

Globally, the number of newborn deaths was 5 million in 1999 and it decreased to 2.5 million in 2017 [2]. More than 40% of all deaths in children younger than five years of age occurred during the neonatal period [3]. The 2016 Ethiopian Demographic Health Survey (EDHS) reported one in every 35 children died within the first months of life [2].

Newborns are the most vulnerable children and many conditions that result in a newborn death can easily be prevented by providing a combined approach to the mother and her baby during her pregnancy, delivery, and effective care after birth [4].

Infection, cord strangulation, and asphyxia are the common causes of neonatal deaths [5]. Although different studies were conducted to assess the magnitude and associated factors of neonatal sepsis in Ethiopia [4–13], the findings were inconsistent and varied. As documented in different kinds of literature, most frequently factors caused neonatal sepsis were low birth weight, preterm birth, maternal infection, prolonged labor, prolonged rupture of membrane, a complication of pregnancy and instrumental delivery [6–12]. Other like asphyxia [4], size of the neonate at birth and neonatal care practice, number of pregnancy, maternal morbidity were less frequently cause neonatal sepsis [9].

There are fragmented studies that were done to estimate the magnitude of neonatal sepsis and associated factors in Ethiopia. However, the prevalence of neonatal sepsis ranges from 78.6%, in Shashemene, Oromia region [6] to 23.8% in Bahir Dar [12]. It showed that there is a high discrepancy of study findings and there was no nationwide study that represents the pooled prevalence of neonatal sepsis in Ethiopia. Thus, the purpose of this study was to estimate the pooled prevalence of neonatal sepsis and its association with birth weight and gestational age in Ethiopia.

## Methods

### Search approach and appraisal of studies

The Preferred Reporting Items for Systemic Reviews and Meta-analysis (PRISMA) guideline was followed for this systematic review and meta-analysis [14]. Studies that addressed neonatal sepsis and its association with birth weight and gestational age in Ethiopia were included. EMBASE, PubMed, Google Scholar, CINHAL and Cochrane library were searched. The strategy of searching was carried out using the following searching terms like: “prevalence”, “neonatal sepsis”, “NICU”, hospital, < 28 days of birth, and “Ethiopia”. The search terms were used in combination and separately using Boolean operators “AND” or OR “” or AND,NOT or AND, NOT. Endnote reference manager software was utilized.

### Inclusion and exclusion criteria

Studies were eligible for inclusion if they: (1) were done in regards neonatal sepsis and its associations with birth weight and gestational age in Ethiopia, with the design of cross-sectional and a cohort studies; (2) reported with the English language; (3) No restrictions were placed on study settings. Studies were excluded if they are single case study design or qualitative study and were published in a book, or report.

### Measuring outcome variables

The outcome of this review was prevalence of neonatal sepsis among admitted newborns. Neonatal sepsis is defined as neonates with presence of at least one clinical sign plus at least two laboratory results which are suggestive for neonatal sepsis (WBC, ESR, Platelet count, CRP, ANC, and Blood glucose) or neonates who are diagnosed as sepsis by attending physician and fulfill sepsis criteria within 0–28 days of life. The second outcome of the study was to determine the association between neonatal sepsis with birth weight and gestational age.

### Study screening and selection

Two independent reviewers extracted the data by using the standard format which includes year of publication, primary author, study design, study setting, sample size, a region of the study, response rate and prevalence of neonatal sepsis. They independently carried out data extractions and article inclusions. Disagreements of article inclusion were resolved with discussion and consensus. Studies that met the Newcastle-Ottawa Scale tool, criteria in terms of enough sample size, clarity of research aims, appropriateness of design, recruitment, data collection, analysis and reporting results were included in the review. Then the full-text of potentially eligible papers assessed against the inclusion criteria. The relevance of the reviewed studies checked based on their topic, objectives, and methodology. Newcastle-Ottawa Scale was used to give a score for articles (NOS) [15, 16]. Finally, articles with a score of ≥6 were included in the final analysis.

### Statistical analysis

Microsoft Excel spreadsheet and Stata version 14 software were used to extract and analyzed the data, respectively. I^2^ statistics test was used to quantify heterogeneity among studies [17]. Laird’s random Effects model was used to estimate the pooled prevalence of neonatal sepsis. Subgroup analysis was done by study setting to minimize the random variations between the point estimates of the primary studies. Furthermore, univariate meta-regression analysis was undertaken by sample size, study design, region, and publication date. The association between neonatal sepsis infection with birth weight and gestational age were determined with odds ratio.

### Publication bias

Begg and Egger’s test of the intercept and the funnel plot of precision asymmetry used to detect publication bias [18].

## Results

**Fig. 1:**
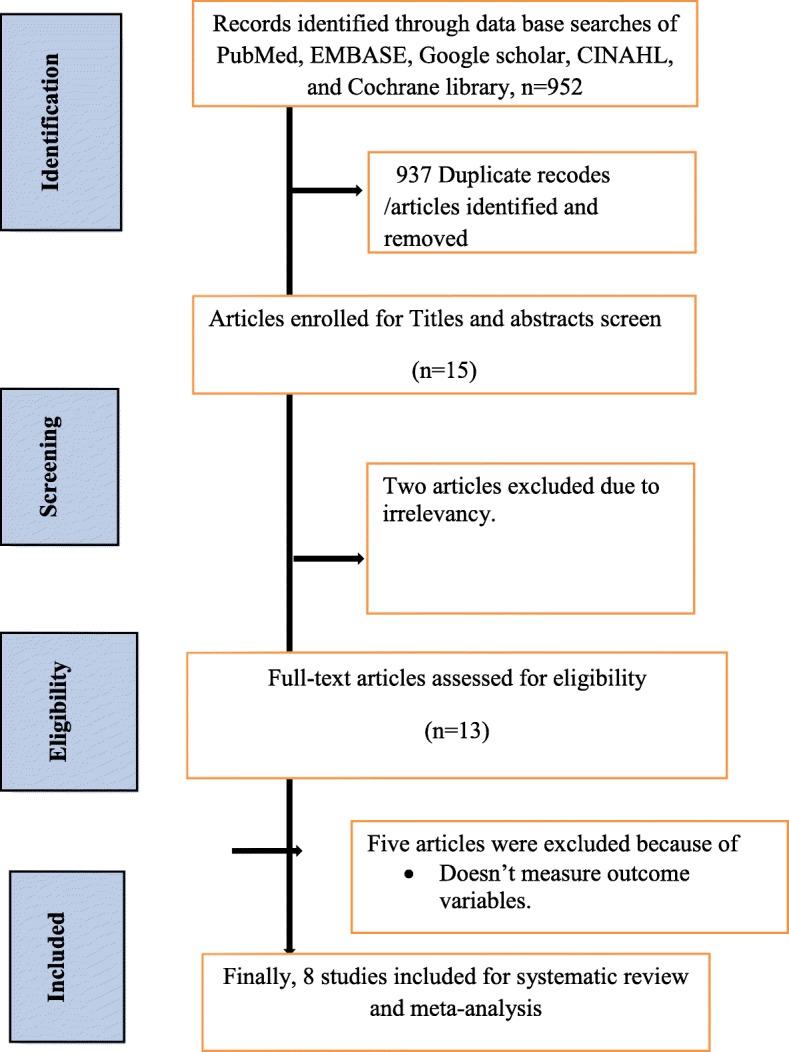
Flowchart diagram to reflect the selection of studies for systematic review and meta-analysis of the prevalence of neonatal sepsis and its association with birth weight and gestational age in Ethiopia

About 952 articles regarding neonatal sepsis were retrieved in Google scholar, PubMed, EMBASE, CINHAL, and Cochrane and using other databases. Among them, 939 were excluded due to duplications and irrelevancies, 13 articles were enrolled for abstract and title screen. Thirteen studies fulfilled the eligibility criteria and from them, five studies were excluded since they did not report the outcome variable [11, 19–22]. Finally, 8 studies were included in this systematic review and meta-analysis (Fig. 1).

### Characteristics of original studies

As described below Table 1, these 8 studies were published from 2014 to 2018. In the current systematic review and meta-analysis, 9032 live births were involved to estimate the pooled prevalence of neonatal sepsis among newborns. Both cross-sectional and cohort study designs were included in this study. The highest prevalence (77.8%) of neonatal sepsis was reported in Gonder Town, Ethiopia [6] while the lowest prevalence (23.8%) was reported in Bahir Dar, Amhara region [12]. In this meta-analysis, three regions of the country were represented; two studies were from the Amhara region [6, 12], one study was from Tigray [23] and five studies from the Oromia region [7–10, 13]. Six studies were conducted using a cross-sectional study design while two were cohort studies (Table 1).

**Table 1:** Descriptive summary of 8 studies included in the meta-analysis of the prevalence of neonatal sepsis in Ethiopia, 2018

Authors /Year of publication	Region	Study setting	Study design	Response rate	Sample size	Out-come	Prevalence
Demessie AG et al. 2017 [6]	Amhara	Institutional based	cross sectional	100%	769	522	67.9
Getablew A et al. 2018 [7]	Oromiya	Institutional based	cross sectional	100%	244	190	77.86
Tewabe T et al. 2018 [12]	Amhara	Institutional based	cross sectional	100%	391	93	23.8
Woldu MA et al. 2017 [8]	Oromiya	Institutional based	cross sectional	100%	306	229	74.83
Roba et al. 2017 [10]	Oromiya	Institutional based	cross sectional	100%	3418	1207	35.31
Sime H et al. 2014 [13]	Oromiya	Institutional based	Cross sectional		225	90	40
Gerenesea H et al. 2017 [23]	Tigray	Institutional based	Cross sectional	100%	246	49	22.7
Debelew GT et al 2014 [9]	Oromiya	Institutional based	cohort	100%	3463	1188	34.3

### Publication bias

Begg and Egger’s test showed that there is no publication bias with a *p*-value of (*p* = 1) and (*p* = 0.615), respectively. The Egger’s test of the intercepts (B0) was 0.089(CI -0.322, 0.50).

### Meta-analysis

**Fig. 2:**
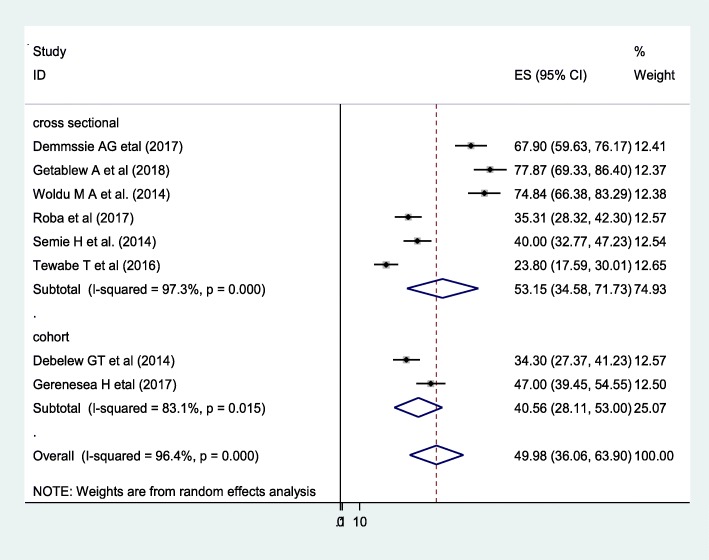
Subgroup analysis and prevalence of neonatal sepsis in Ethiopia, 2018(*n* = 8)

In this study, the pooled prevalence of neonatal sepsis was 49.98% with a confidence interval of (CI: 36.06, 63.99). A random-effect model was employed to estimate the pooled prevalence of neonatal sepsis due to severe heterogeneity (I^2^ 96.4, *p*-value = 0.000) was observed between the studies. Furthermore, subgroup analysis was done by region, sample size, publication year and study design but none of them were significant (Fig. 2).

### Meta regressions

Univariate meta-regression analysis was used to investigate the source of variation between studies by study design, publication date, sample size, region, and study design but none of them were statistically significant (Table 2). Furthermore, a sensitivity analysis was carried out and all studies were under the confidence intervals.

**Table 2:** Univariate Meta-regression analysis of studies on neonatal sepsis infections in Ethiopia, 2018

		Co-efficient	*P* value
Publication year		1.3	0.911
Sample size		0.01	0.9
Region	Amhara	−23.8	0.6
	Oromiya	−12.7	0.74
	Tigray	1	
Study design	Cross sectional	1	
	Cohort	−22.7	0.46

### Association between birth weight and neonatal sepsis

**Fig. 3:**
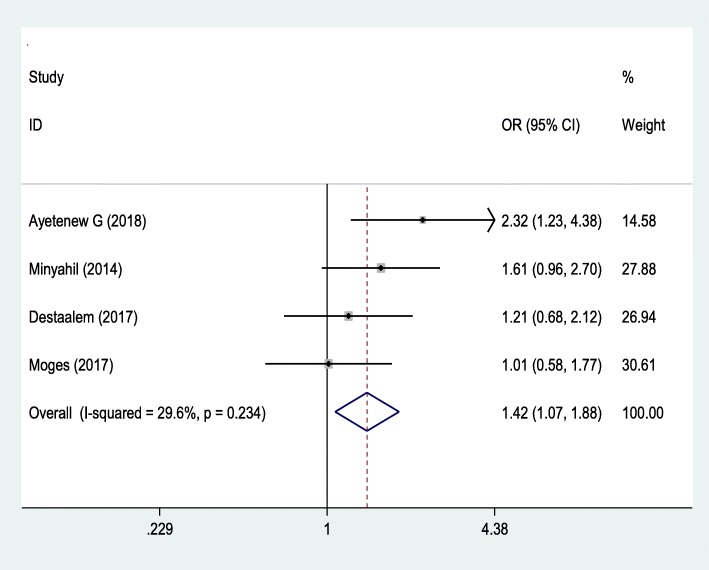
The pooled odds ratio of the association between birth weight and the occurrence of neonatal sepsis in Ethiopia

Four studies [6–8, 22] were used to examine the association between neonatal sepsis with birth weight (Fig. 3). The analysis revealed that neonatal sepsis was significantly associated with the low birth weight with OR 1.42 (95% CI, 1.07, 1.88). This meta-analysis showed that the odds ratio of low birth weight was 1.42 times to develop neonatal sepsis than normal-weight born babies. The Begg and Egger tests showed that there was no publication bias with the *p*-value of (*P* = 0.497) and (*p* = 0.076), respectively. The Egger test with the Bo = 2.797 (− 0.722, 6.32).

### Association between gestational age and neonatal sepsis

**Fig. 4:**
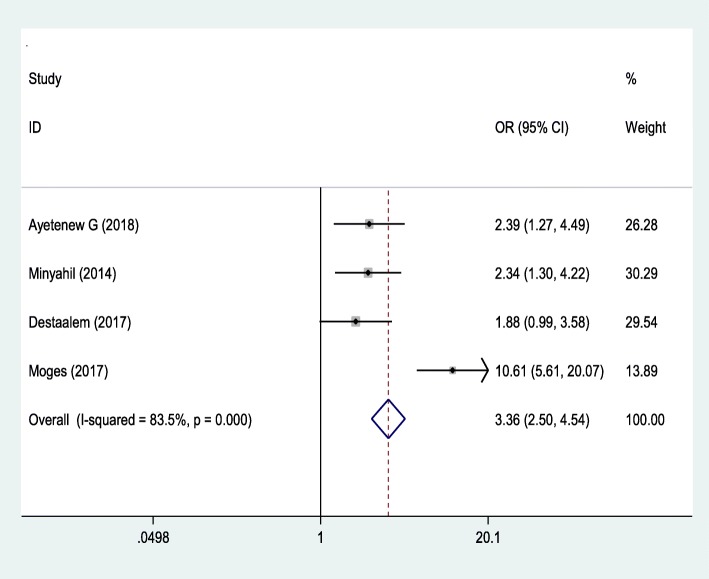
The pooled odds ratio of the association between gestational age and occurrence of neonatal sepsis in Ethiopia

The third outcome of this study was to determine the association between gestational age and neonatal sepsis. The analysis of these four studies [6–8, 22] revealed that neonatal sepsis was significantly associated with the gestational age of newborn with OR 3.36 (95% CI: 2.50, 4.54). Preterm babies were 3.36 more likely to develop neonatal sepsis than term babies. The Begg and Egger tests showed that there was no publication bias with *P*-value (P = 0.497) and (p = 0.076), respectively. The Egger test with the Bo = 2.797 (− 0.722, 6.32) (Fig. 4).

## Discussion

This systematic review and meta-analysis showed the pooled prevalence of neonatal sepsis and its association with birth weight and gestational age among admitted neonates. The pooled prevalence of neonatal sepsis among newborns in the first 28 days of life was 49.98% (CI: 36.06, 63.90). The findings of this review was similar to the global report of the burden of pediatric and neonatal sepsis, 48% [24], but it is higher than studies done in; Kenya, 23.9% [25], Nigeria, 18.2/1000 live births [26], India, 7.6% [27], Temeke and Mwananyamala hospitals, Tanzania, 31.4% [28], and Egypt, 45.9% [29]. This may be due to difference in socio-demographic characteristics of respondents, diagnostic modalities, low antenatal care visits in the country, which is 62% and only 26% of mothers gave birth in health facilities and the high prevalence of low birth weight and preterm births [4] compared to other countries. Because of this, the prevalence of neonatal sepsis is high in this study compared to other studies. Even though the Ethiopian government implements different strategies to reduce early neonatal infections, still the burden is prevalent and further work is needed to reduce deaths related to newborn sepsis. Therefore, improving antenatal service usage and encouraging mothers to give birth at health institutions will help to reduce early neonatal sepsis especially for low birth and preterm deliveries.

The current studies finding also high compared to systematic review and meta-analysis done in developed countries, 17.2% [30]. This huge difference may be due to lack of health facilities, early diagnostic or screen modalities and prompt treatment actions between this study and developed countries.

Based on subgroup analysis, the higher pooled prevalence of neonatal sepsis was found from cross-sectional studies (53.15%) compared to cohort studies (40.56%). The possible explanation is since the cross-sectional study is one spot study and the prevalence may be high due to seasonal variations.

Neonatal sepsis was influenced by different factors. Preterm newborn babies were 3.36 times more likely to develop neonatal sepsis compared to term newborns. This finding is in line with studies done in Tanzania [28], USA [29, 31] and China [32]. The possible explanation is that preterm babies have immature immune systems (low neutrophil storages) and body organs that fight infections. Due to this, when health professional undergoing procedures like invasive treatment and may be exposed to nosocomial infection, they are more likely prone to develop neonatal infections. There is rapid exhaustion of bone marrow reserve during sepsis. Nowadays immune replacement therapies are widely explored for correcting the immune deficiencies of preterm, and it prevents neonatal infections. Therefore, staff training and education about infection prevention is a crucial step to prevent nosocomial infections.

The birth weight of the newborn was one of the determining factors for neonatal sepsis. Newborns with less than 2.5 kg were 1.42 times more likely to develop neonatal sepsis than newborns born with 2.5 kg and above. This finding is consistent with studies done in Afghanistan [33], Sweden [34] and Spain [35]. This may be due to low birth weight newborns are mostly premature, have immature immune system, unable to feed, easily lose their heat, low store of glucose and more likely risk to develop hypoglycemia may increases the likelihood of neonatal infections.

### Limitation

Since it is a first systematic review, lack of enough literature, and use odds ratio to estimate the predictor variables may be affected by other confounding variables. Limited available of studies and low sample size might affect the pooled prevalence.

## Conclusion

The pooled prevalence of neonatal sepsis in Ethiopia was high. Therefore, it is recommended to have more skilled health personnel and advanced equipment while providing maternal and neonatal health care services. Health care providers should adhere to aseptic precautions while performing procedures, especially on low birth weight and preterm infants.

## Data Availability

Identifying/confidential patient data should not be shared.
